# Heidelberg Letter

**Published:** 1882-04

**Authors:** W. H. Fitch

**Affiliations:** Heidlberg


### ARTICLE V.

HEIDLBERG, FEBRUARY, 1882.

EDITORS MEDICAL JOURNAL AND EXAMINER: — One who spends any considerable time abroad studying medicine or visiting hospitals, and fails to visit Heidelberg, will miss one of the most essential ingredients of his feast. This place has been rich in medical glory so long as to impregnate the air with its sanctity, and you breathe inspiration before you go to the hospital to see what manner of men the professors are. Although the inhabitants are not very numerous—about twenty-five thousand — yet for hospital advantages it is no mean city, having about four hundred and fifty-three beds for patients, and at present more are knocking for admission than can be accommodated. The larger part of the hospital consists of detached buildings that contain but few beds, and large windows, so that the supply of sunlight and air surpasses any similar buildings I have happened to particularly observe, except the new hospital at Genoa. But I came here particularly to see a former Vienna acquaintance, Prof. Czerny, who, still quite a young man, not over forty, and probably not as old, has made a reputation world-wide for his bold, skillful and successful operations, and every day adds new luster to his fame. Formerly an assistant of Prof. Billroth, of Vienna, ten years ago he went to Fribourg and stayed five years, then came here to succeed the celebrated Simon, and although a comparatively small man, fills the chair full.

The opportunity for studying surgery here is almost unsurpassed, except for injuries, even by the large cities. The number of students is comparatively small, so that each can see and hear all that is said and done, while the operator is not hid by a crowd of assistants, as in Berlin or Vienna. But, to better illustrate, I will give you an epitome of his clinic for a day, which seemed to be only an average one. At each clinic one or more students are called down to examine the patients, give their diagnosis, prognosis and treatment, and defend the same before the class. In this way they become practical surgeons at the beginning of their career, instead of the end of it.

*Case* I. Æt. forty; strumous diathesis; has hydrocele on left side. Parts shaved, and washed with carbolic lotion. When scrotum was opened and fluid evacuated, the testicle was found somewhat enlarged, and tunica vaginalis much thickened, so the testicle was removed, and found studded with caseous deposits. Vessels were tied with catgut, drainage tube inserted, and parts brought together with sutures. Had it not been determined to remove the testicle, a deep, loose suture would have been taken, and parts allowed to heal from the bottom.

*Case* II.—A young girl of eleven was presented, who had lost her nose from lupus, and had the defect remedied by a plastic operation, the flap being turned down from the forehead. The face was covered with eczema, for which ung. zinci oxidi was ordered.

*Case* III.—A female, æt. about thirty-six, from whom, a few weeks previous, the professor had removed the uterus for malignant disease, and now removed the last silver suture. The professor has operated ten times, with six recoveries and four deaths; always operates through the vagina. Patient was ordered home, and to report in six weeks. The success in this class of operations promises much to the sufferers from this malady, when taken early.

*Case* IV.—Female, æt. sixty-five, was presented, on whom an operation had been performed for epithelioma of left frontal region, that had extended through the cranium and affected the dura mater. The diseased tissues had been removed, including a portion of the dura mater, and wound covered by a flap from scalp. The wound was now entirely healed, and pulsations of brain could be seen through scalp covering the aperture in the skull. Patient dismissed, to report in a few weeks.

*Case* V.—Child had been burned over entire anterior part of chest. Skin-grafting had been necessary, for which pieces of skin as large as thumb nail had been taken from an amputated leg, and successfully transplanted. Wound or burn was now nearly healed.

*Case* VII.—Male, with enlarged lymphatic glands in submaxillary region, which were injected with 50 per cent. solution of iodoform. Several patients similarly affected were undergoing like treatment, but had not been under observation long enough to furnish definite results.

*Case* VIII.—Female ambulant. Had complained for two years of dysphagia. Had not lost flesh; general health good as usual; menstruation normal; nervous system undisturbed. Could swallow liquids without trouble. Solids would apparently go just below the larynx and return. Sometimes they would be swallowed after several unsuccesful attempts. Swallowing was not accompanied with pain, except the choking. Patient was given some bread to eat, and demonstrated the difficulty several times. Swallowing solids was sometimes facilitated by taking liquids. It might be stricture, but there had been no cause for stricture; moreover, a bougie passed down the œsophagus proved the absence of stricture. It might be malignant disease, but none could be felt; the bougie or regurgitated food was not tinged with blood; besides, its duration almost excluded the disease. It might be nervous, but no impairment of the nervous system could be observed or elicited, although a longer observation might be necessary to settle that point. It might be a cul de sac into which food deviated, but none could be felt in an abnormal position after swallowing. It might be owing to pressure on the parts by enlargement of thyroid gland. This might even be the case when the gland did not seem to be perceptibly enlarged, as it often pressed in between trachea and œsophagus, and sometimes behind both, and eluded detection. In the present case, the gland was somewhat enlarged on right side, but only to a slight extent; in fact, so little as to hardly attract attention; nevertheless, the lecturer had had one case similar to the present, in which he removed the gland with perfect relief. The patient was ordered iodide of potassium three times a day, and emplastrum mercurial. Should there be no alleviation of symptoms in due time, and nothing else discoverable, the gland would be extirpated; or should a cul de sac be found, it would be remedied by removal.

*Case* IX.—Young man of seventeen, with cleft palate. Operation for closure was performed under chloroform, with head in dependent position. Edges of cleft were denuded with scissors and knife, and brought together with silver and catgut sutures. Incisions were made on either side of and parallel to the cleft, and tissues loosened from hard palate to relieve tension on sutures. Difficulties of this position are profuse hæmorrhage, which obscures the operation, and œdema of brain, which is liable to occur in adults.

*Case* X.—Young woman upon whom Battey's operation had been performed three weeks previously, for hystero-epilepsy. Wound had entirely healed, but there was some tenderness in right groin, with elevation of temperature, and abscess feared. Treatment was expectant. Spasmodic manifestations had disappeared since the operation.

This is only a sample, detailed in brief, of the clinical lectures and operations that last from one and a half to three hours. Students are allowed to perform some of the operations, under the direction of the professor. One of the students did the castration detailed above. Dr. Czerny talks English very well, and welcomes strangers with cordiality. Some of his private patients are taken into the hospital. Patients come from the United States to consult him concerning maladies to which he has given special attention, and for operations in which he has become particularly skilled.

From the surgical clinic those who desire go to the ophthalmic clinic and lecture of Prof. Becker, where abundant material is found, and numberless cases full of scientific instruments, charts, calculations, etc., for determination of refractions, and other intricate and difficult problems connected with the eye. And so I might enumerate various other departments, but enough has been given to illustrate something of what may claim the attention at Heidelberg. W. H. FITCH, M.D.

---

ANÆSTHETIC MIXTURES.—The Vienna mixture, with which eight thousand operations have been performed without an accident, consists of ether, three parts; chloroform, one part. Billroth's favorite anæsthetic mixture is chloroform, three parts; ether, one part; alcohol, one part. An English mixture, known as the A. C. E. mixture, consists of alcohol, one part; chloroform, two parts, ether, three parts.

Owing to the different volatility and specific gravity of the different anæsthetic liquids, the vapors have, necessarily, a different composition from that of the mixtures themselves. The value of a mixture must, therefore, in part, be determined empirically. Some experiments have been made in the mixing of heart-stimulants with chloroform. Sanford mixed one pound of chloroform with two drachms and a half of amyl nitrite. Others have added oil of turpentine to the chloroform. The objection so far has been that such mixtures cause a headache.—*Med. Record.*

